# Neutralizing antibodies levels are increased in individuals with heterologous vaccination and hybrid immunity with Ad5-nCoV in the north of Mexico

**DOI:** 10.1371/journal.pone.0269032

**Published:** 2022-06-24

**Authors:** Karla Cervantes-Luevano, Astrid N. Espino-Vazquez, Gonzalo Flores-Acosta, Johanna Bernaldez-Sarabia, Olivia Cabanillas-Bernal, Jahaziel Gasperin-Bulbarela, Ricardo Gonzalez-Sanchez, Andreu Comas-Garcia, Alexei F. Licea-Navarro

**Affiliations:** 1 Department of Biomedical Innovation, Center for Scientific Research and Higher Education at Ensenada, Baja California, Mexico; 2 Facultad de Medicina y Centro de Investigación en Ciencias de la Salud y Biomedicina, UASLP, San Luis Potosi, Mexico; Instituto Butantan, BRAZIL

## Abstract

The coordinated efforts to stop the spread of the severe acute respiratory syndrome coronavirus 2 (SARS-CoV2) include massive immunization of the population at a global scale. The humoral immunity against COVID-19 is conferred by neutralizing antibodies (NAbs) that occur during the post-infection period and upon vaccination. Here, we provide robust data showing that potent neutralizing antibodies are induced in convalescent patients of SARS-CoV-2 infection who have been immunized with different types of vaccines, and patients with no previous history of COVID-19 immunized with a mixed vaccination schedule regardless of the previous infection. More importantly, we showed that a heterologous prime-boost in individuals with Ad5-nCoV (Cansino) vaccine induces higher NAbs levels in comparison to a single vaccination scheme alone.

## Introduction

According to the World Health Organization (WHO), from January 2020 until October 2021, in Mexico, there have been 3,767,758 confirmed cases of Coronavirus disease (COVID-19) with 285,347 lethal cases, this places Mexico as fourth place in cumulative deaths, only surpassed by USA, Brazil, and India [1]. From December 2020 to this date, eight vaccines in phase III or IV of preclinical studies have been approved for use in Mexico; these include BNT162b2 (Pfizer, Inc,/BioNTech); AZD1222 Covishield (AstraZeneca); CoronaVac (Sinovac); Ad5-nCoV Covidecia (Cansino); BBV152 Covaxin (Bharat Biotech International Limited); Ad2-6.COV2-S (Janssen-Cilag); Gam-COVID-Vac (Sputnik V); BBIBP-CorV (Sinopharm), and recently mRNA-1273 (Moderna); only Cansino and Janssen-Cilag have a phase III clinical study in Mexico. Since January 2021, 179,063,050 doses have been applied and a total of 107,031,525 individuals have been vaccinated in the Mexico [2]. Several reports have presented convincing evidence that is suggestive that a mixture of vaccines could increase the immune response against the severe acute respiratory syndrome coronavirus-2 (SARS-CoV-2). However, these combinations have not included the Cansino vaccine as the primary immunogen. This study aimed to evaluate the humoral immunity against SARS-CoV-2 in a population consisting of unvaccinated convalescents, immunized individuals with a single (complete and incomplete) vaccination scheme, and individuals with a mixed vaccination scheme, to explore differences by sex, age, vaccines delivery technology, immunization schemes, and previous infection, analyzing neutralizing antibody (NAb) response either as a categorical (positive/negative) or continuous variable (NT50). Furthermore, we focused our analysis on testing positive sera against four SARS-CoV-2 variants: original (D614G), alpha (B.1.1.7), delta (B.1.617.2), and epsilon (B.1.429). To our knowledge, this is the first report of neutralizing antibodies in individuals previously infected and with two doses of Pfizer vaccine in combination with CanSino, and CanSino with Janssen-Cilag.

## Methods

### Clinical samples collection, handling and storage

Sera from convalescent individuals were collected from 30 days up to 6 months post-symptom onset. Plasmas from the first dose of BNT162b2 were collected on day 21 until day 42, post-immunization. Plasmas from completed vaccination schemes: BNT162b2, mRNA-1273, Ad5-nCoV, Ad26.COV2-S and CoronaVac were collected 21 to 90 days post-complete scheduled immunization. On average, plasmas from mixed schedules were collected 21–60 days post-immunization of complete vaccine doses. Blood was drawn in EDTA-treated tubes at the CICESE facilities, with the written consent of patients under the General Hospital of Ensenada ethics committee approved protocols (HGE-21-17). None of the donors had a history of hospitalization for COVID-19.

After collection, blood was centrifuged at 1,500 rpm for 15 min, and plasma was separated from the cellular fraction and heat-inactivated at 56°C for 30 min. Samples were kept at 4°C until complete analysis.

### Cell culture, viral strains isolation and virus amplification

Vero E6 cells were maintained in DMEM media supplemented with 2 mM L‐Glutamine, 10% FBS, and 100 units/mL penicillin-streptomycin in a 5% CO_2_ humidified incubator. For virus isolation, nasopharyngeal swabs positive for SARS-CoV-2 infection were used for infection in 2% FBS DMEM supplemented media. Detection of SARS-CoV-2 was performed by qPCR using Logix Smart^™^ Coronavirus 2019 (COVID-19) Test, considering a threshold of 18–25 Cts for selection. After 2 days post-inoculation, the cytopathic effect (CPE) was evident and the harvest of viral lysates was performed on day 3 post-inoculation. The supernatant was collected and centrifuged at 14,000rpm 4°C to eliminate debris; aliquots of the virus were snap-freeze and kept in liquid nitrogen for future use in neutralization analysis. One hundred μL of viral lysate was used for total nucleic acid extraction for the confirmatory testing of SARS-CoV-2 by qPCR and sequencing. The virus was titrated in serial 1-log dilutions (from 1 log to 9 logs) to obtain a 50% tissue culture infective dose (TCID_50_) on a 96‐well culture plate of Vero E6 cells.

### CPE based assay for neutralizing antibodies

Titration of neutralizing antibodies was performed as previously described [3] with minor modifications. Briefly, Vero E6 cells (1.5×10^4^) were seeded in a 96-well Clear Flat Bottom TC plate in DMEM 2% FBS 24h prior to infection. HI-plasma samples were two-fold serial diluted starting 1:40–1:10,240; dilutions of plasma were mixed with an equal volume of viral solution containing 0.02-TCID_50_ SARS-CoV-2 per cell. The plasma-virus mixture was incubated for 1h at 37°C with 5% CO_2_, and then 100μL of the mixture was transferred to a semi-confluent Vero E6 monolayer. Plates were incubated for 96 h in the described conditions. Analysis of CPE prevention by neutralizing antibodies was performed by neutral red uptake assay, and the NT_50_ titer was calculated in GraphPad prism 9.

### Whole-genome sequencing

For complete genome sequencing, viral RNA was isolated from culture supernatant with the kit QIAamp Viral RNA Mini Kit according to the manufacturer’s instruction. Depletion of ribosomal RNA and cDNA synthesis was performed using Zymo-Seq RiboFree universal cDNA according to protocol recommended by the manufacturer. After six rounds of PCR, the samples were analyzed in a Bioanalyzer (Agilent Technologies, Santa Clara, California, USA), and the libraries were constructed using the Ion Total RNA-Seq Kit v2. After that, samples were quantified, pooled, and sequenced in two 530 chips using Ion Torrent technology in an S5 NGS sequencer (Thermo Fisher Scientific, Carlsbad, California, USA). A nearly full-length viral contig was obtained in each sample and was compared in identity to the hCov-19/Wuhan/WIV04/2019. Complete sequences were deposited in GISAID under the accession numbers: EPI_ISL_747242 (D614G variant); EPI_ISL_2455244 (epsilon variant); EPI_ISL_2249247 (delta variant) and EPI_ISL_2455241 (alpha variant).

### Statistical analysis

Statistical analyses were done in R for Windows version 4.0.5 and IBM SPSS Statistics version 26.0.0. NAb production was evaluated as a continuous variable (Log_10_NT_50_) and for some analyses, it was also modeled as categorically defining NT_50_ > 0 as a positive result and NT_50_ = 0 as a negative result. Differences between frequencies of positive and negative results were evaluated using the Chi-squared test (*X*^2^) (confidence level 95%). To test normality in distributions of NT_50_ and Log_10_NT_50_, we used the Shapiro–Wilk normality test (confidence level 95%) and Q-Q plots. The correlation between age and Log_10_NT_50_ was determined using the Pearson correlation coefficient. Comparisons for categorical variables with two possible values, such as sex, convalescents/not infected, or 1-dose/2-doses of vaccine, were performed using the Mann–Whitney U test (confidence level 95%). Distributions of Log_10_NT_50_ among vaccines and SARS-CoV-2 variants were compared using the Kruskal–Wallis test followed by Dunn’s multiple comparison test (confidence level 95% for both tests), with Bonferroni adjusted alpha level. Effect sizes for statistical tests were obtained using the R package ‘effect size’ version 0.4.5 [4], where rank biserial (r) was estimated for Mann-Whitney U tests, and the rank epsilon-squared (ε^2^) for Kruskal–Wallis tests, following the Package interpretation guidelines. Phi (ϕ) and Cramer’s V were estimated as size effect for the *X*^2^ test, following the interpretation of [5].

## Results

### General characteristics of the studied population

A total of 266 samples were collected from the 247 volunteers. The median age was 43 years (range 19–82 years) with 63.16% of females (n = 156) and 36.84% of males (n = 91). From the vaccinated group 68.04% (n = 181) had no history of previous SARS-CoV-2 infection while 26.31% (n = 70) had a previous qPCR positive result and no history of hospitalization for SARS-CoV-2, 3.75% (n = 10) were from unvaccinated convalescent, and 1.87% (5) were unaware of Covid infection. For these vaccinated individuals, 43.75% (n = 112) corresponded to Ad5-nCoV, 22.66% (n = 58) to BNT162b2 (9 with 1-dose and 49 with 2-doses); 14.84% (n = 38) to Ad26.COV2.S; 7.42% (n = 19) to CoronaVac; 3.13% (n = 8) to mRNA-1273, and 8.2% (n = 21) had a combination of two vaccines schedules (mixed vaccines).

### Neutralizing antibodies levels among studied individuals

Previous studies [6] indicate that in Mexico, the lineages B.1.1.519; B.1; B.1.1.222; B.1.1; B.1.609, and B.1.243 were the most prevalent from March 2020 to March 2021. In a first approach, we measured SARS-CoV-2 neutralizing antibodies as correlates of COVID-19 risk and protection against the B.1 variant MX-BC2/2020 (D614G, GISAID ID: EPI_ISL_747242).

Viral neutralization titers measure the ability of antibodies to prevent viral infection; the CPE-based *in vitro* assay was used to measure the antibodies generated by each participant. The first stage of the study consisted in exploring the effect of age, sex, and a previous SARS-CoV-2 infection on NAb production among vaccinated cases (S1 Fig, S1 Table). A negative but not significant correlation between NAb production and age was observed (Pearson correlation coefficient R (244) = -0.069, *p* = 0.28) (S1C Fig). Sex-based differences in innate and adaptive immunity in SARS-CoV-2 infections seem to be related to the increased risk of intensive care unit admission and overall mortality in men and increased reports of long-Covid-19 symptoms in women [7, 8]. Sex differences have been described in antibody response to vaccines, with a greater outcome of acceptance and greater immune response in females when compared to males in a lifetime with different vaccines [9]. In our study, distributions of NT50 titers were similar for all vaccinated women and men; also, the frequencies of positive or negative results had no association with sex (S1B Fig, S1 Table). Nonetheless, a previous SARS-CoV-2 infection strongly affected the immune response. A history of infection is considered analogous to immune priming; previous reports indicate that vaccination after the first dose of BNT162b2 in recovered individuals increases by a 140-fold peak in antibody titers compared to the pre-vaccine levels [10]. In accordance with other reports [11], in our study, post-vaccination positive results were much more frequent in individuals with a previous infection [*X*^*2*^(1, N = 242) = 17.784867, p = 0.000025, ϕ 0.271093], and their NAb titers were significantly higher than in not infected ones [*U*(N_not_infected_ = 176, N_convalescents_ = 66) = 2270.5, Z = -7.45, *p* = 2.44E-14, r = -0.48] (S1A Fig), indicating that with a history of the previous infection there i’s a boost.

**Fig 1:**
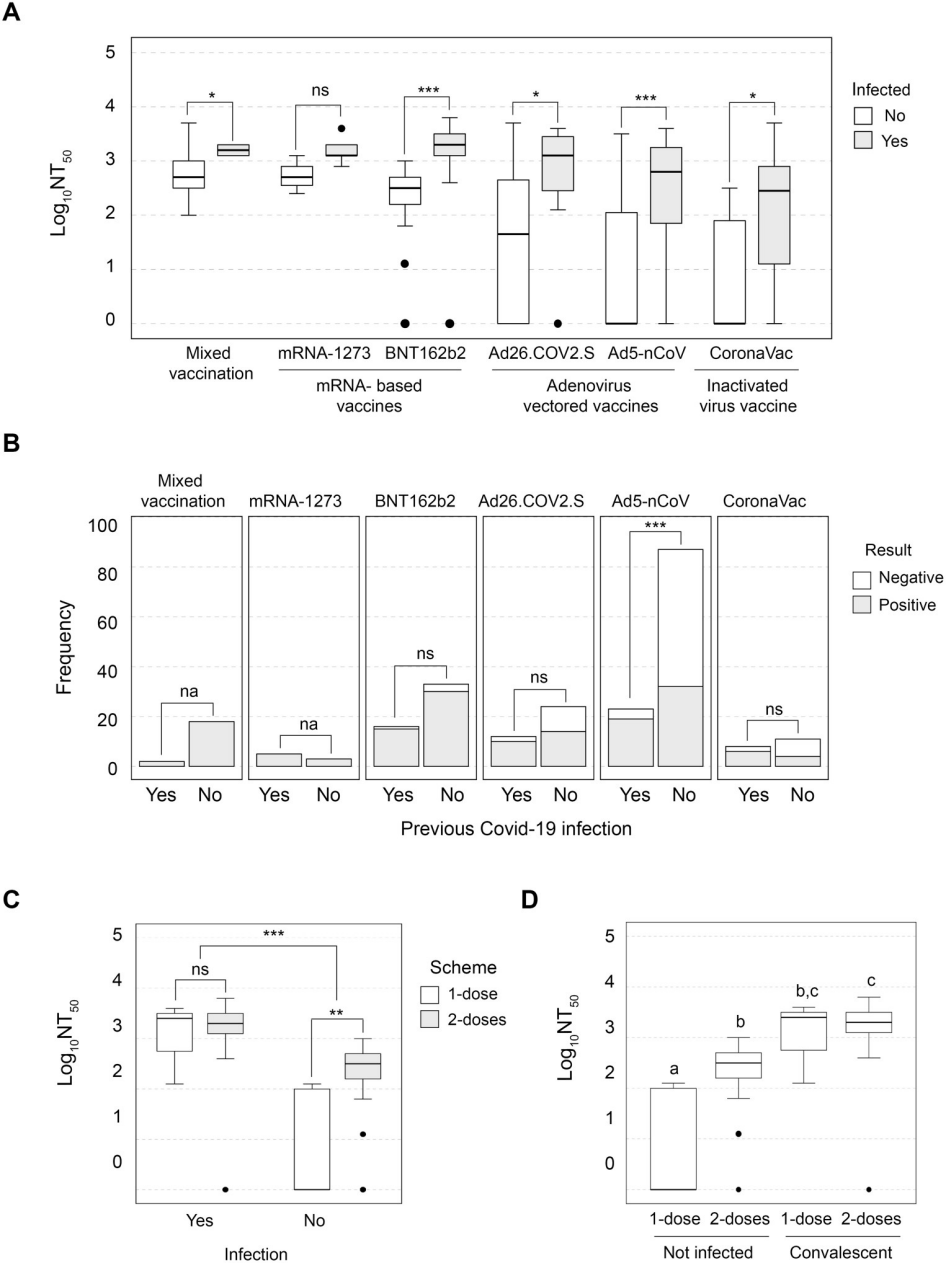
A previous SARS-CoV-2 Infection increases NAb titers in vaccinated people. A) Box plot of NAb titers among vaccines grouped by Covid-19 infection. Pairwise comparisons were performed with Mann-Whitney U test where ns = not significant, * = *p* < 0.05, *** = *p* < 0.001. B) Stacked bars graph of positive and negative results among immunization schemes, grouped by Covid-19 infection. Differences between frequencies were explored by *X*^*2*^ tests, where na = not applicable for the test, ns = not significant, *** = *p* < 0.001. C) Box plot of immunized with 1-dose and 2-doses of BNT162b2, grouped by previous Covid-19 infection. Pairwise comparisons were performed with Mann-Whitney U test where ns = not significant, ** = *p* < 0.01, *** = *p* < 0.001 D) Box plot of convalescent and not infected immunized with 1-dose and 2-doses of BNT162b2 vaccine. All four groups were compared with Kruskal–Wallis test followed by a post hoc Dunn’s test (adjusted alpha = 0.0125). Different letters above bars indicate significant differences between groups (*p* < 0.05), while the same letters denote similar ranks between groups (*p* > 0.05).

The main target for most current SARS-CoV-2 vaccine programs is based on the immune response elicited against the spike protein production by individual. Most studies are therefore focused on measuring virus-specific neutralizing antibodies and/or spike-specific recognizing antibodies, although it is possible that other antibody functions may also contribute to protection [12]. Here, a comparison between vaccines by SARS-CoV-2 infection, showed that the Log_10_NT_50_ ranks from all vaccinated volunteers were higher when a previous infection was registered, but only in the mRNA-1273 vaccine, the difference was not significant although a very large effect was observed [*U*(N_not infected_ = 3, N_convalescents_ = 5) = 2.0, Z = -1.68, *p* = 0.09, r = -0.59] (Fig 1A). In addition, when the association of results and history of infection was tested among vaccines, the two adenovirus-vectored vaccines and the inactivated virus vaccine had a large effect size (Cramer’s V > 0.25) (S1 Table, Fig 1B). However, only for Ad5-nCoV, the difference was statistically different [*X*^*2*^(1, N = 110) = 15.362507, *p* = 0.00009, Cramer’s V = 0.37], possibly due to the effect of the sample size for this group. Volunteers that had no precious SARS-CoV-2 infection who received the Ad5-nCoV vaccine had a significantly higher rate of negative results (63.21%) than convalescents (17.39%).

Differences between the complete and incomplete vaccination schemes (1-dose or 2-doses) of BNT162b2 were also examined, considering a previous SARS-CoV-2 infection (for mRNA-1273 and CoronaVac schemes, we did not have enough cases). Distributions of NAb titers from1-dose and 2-doses of the BNT162b2 vaccine were only different in not previously infected volunteers [*U*(N_2-doses_ = 33, N_1-dose_ = 5) = 18.500000, Z = -2.779749, *p* = 0.003, r = -0.45], as depicted in Fig 1C. Moreover, from all who received the BNT162b2 vaccine, convalescents with the completed scheme produced the highest amount of NAb titers (Fig 1D). Importantly, our results could suggest that the third dose of BNT162b2 for those non-infected individuals may increase protection in the long term.

### Antibody titers are higher in convalescent vaccinated individuals

**Fig 2:**
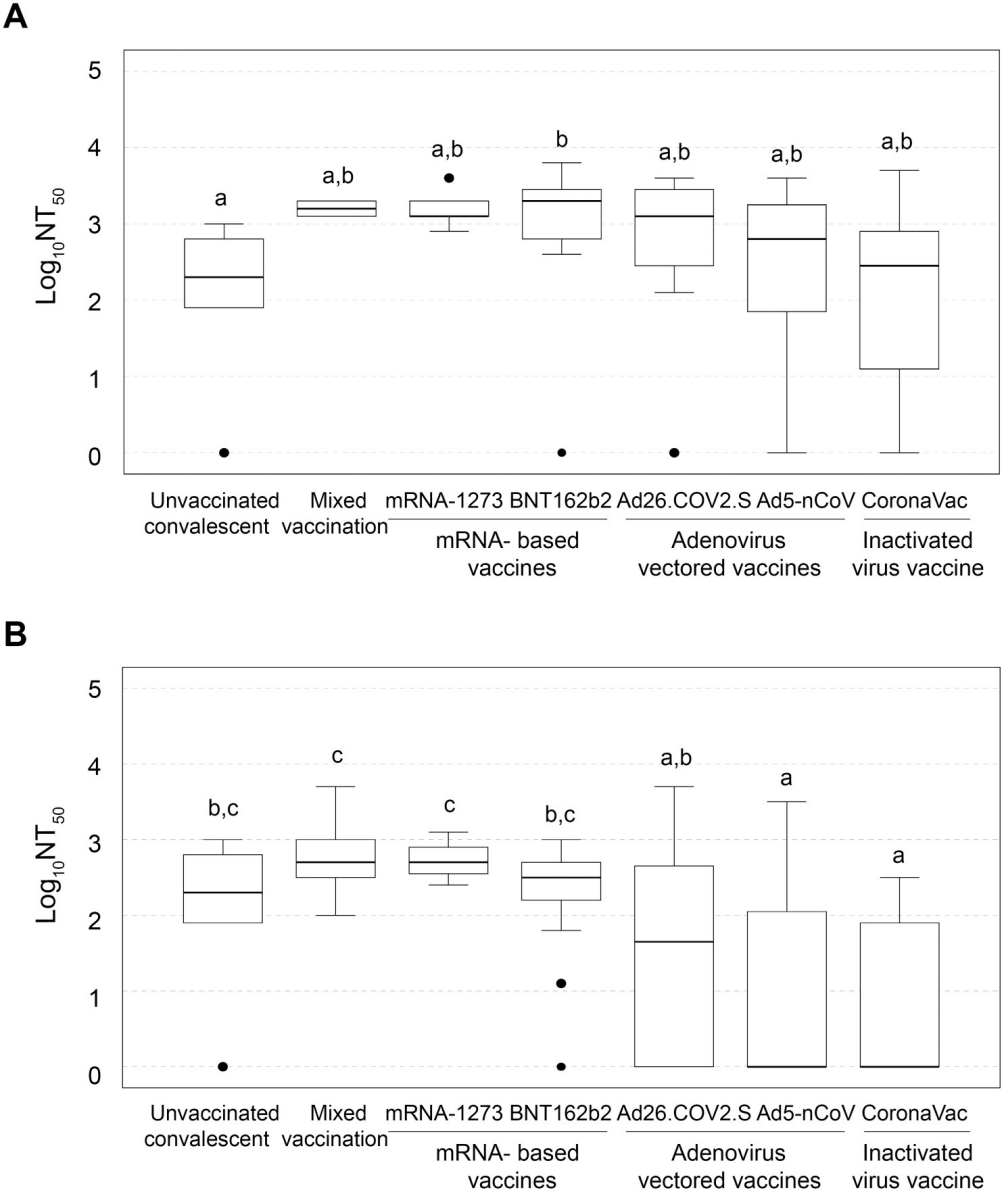
Immunization schemes induce differential NAb production. A) Box plot of NAb titers in convalescents, grouped by immunization scheme. B) Box plot of NAb titers on unvaccinated convalescent and vaccinated not infected, grouped by immunization scheme. The Kruskal–Wallis test compared distributions in immunization schemes; the subsets were established by a post hoc Dunn’s test (adjusted alpha = 0.07). Different letters above bars indicate significant differences between groups (*p* < 0.05), while the same letter denotes similar ranks between groups (*p* > 0.05).

The second stage of the analysis explored differences among types of immunization. In accordance with similar reports, post-vaccination NT_50_ values were higher in all previously infected and vaccinated individuals for all vaccines. The following analysis were conducted separately on the convalescent (N = 76) and not infected (N = 186) data sets. Moreover, an external group of unvaccinated convalescents (N = 68) volunteers was included to contrast natural and artificial immunization. Noticeably, when comparisons among convalescents were done there was no difference between the different immunization schemes and frequencies of positive or negative results [*X*^2^(6, N = 77) = 3.44, p = 0.75, Cramer’s V = 0.21], but when Log_10_NT_50_ was analyzed, significant differences were observed between BNT162b2 and the unvaccinated group [*H*(6) = 16.64, p = 0.01, ε^2^ = 0.56] (Fig 2A). On the other hand individuals that were not infected displayed significant differences among different immunization categories and NAb production by result [*X*^2^(6, N = 186) = 52.59, *p* = 1.41E-9, Cramer’s V = 0.53], and Log_10_NT_50_ levels [*H*(6) = 61.43, *p* = 2.29E-11, ε^2^ = 0.52]. The multiple comparison analysis revealed that mixed vaccination and the complete scheme of mRNA-1273 vaccine induced the highest immunological response, followed by unvaccinated with previous Covid-19 infection and BNT162b2 vaccine. Vaccination with adenovirus-based vaccines and inactivated-viral particles showed the lower NAb ranks in our study (Fig 2B).

**Fig 3:**
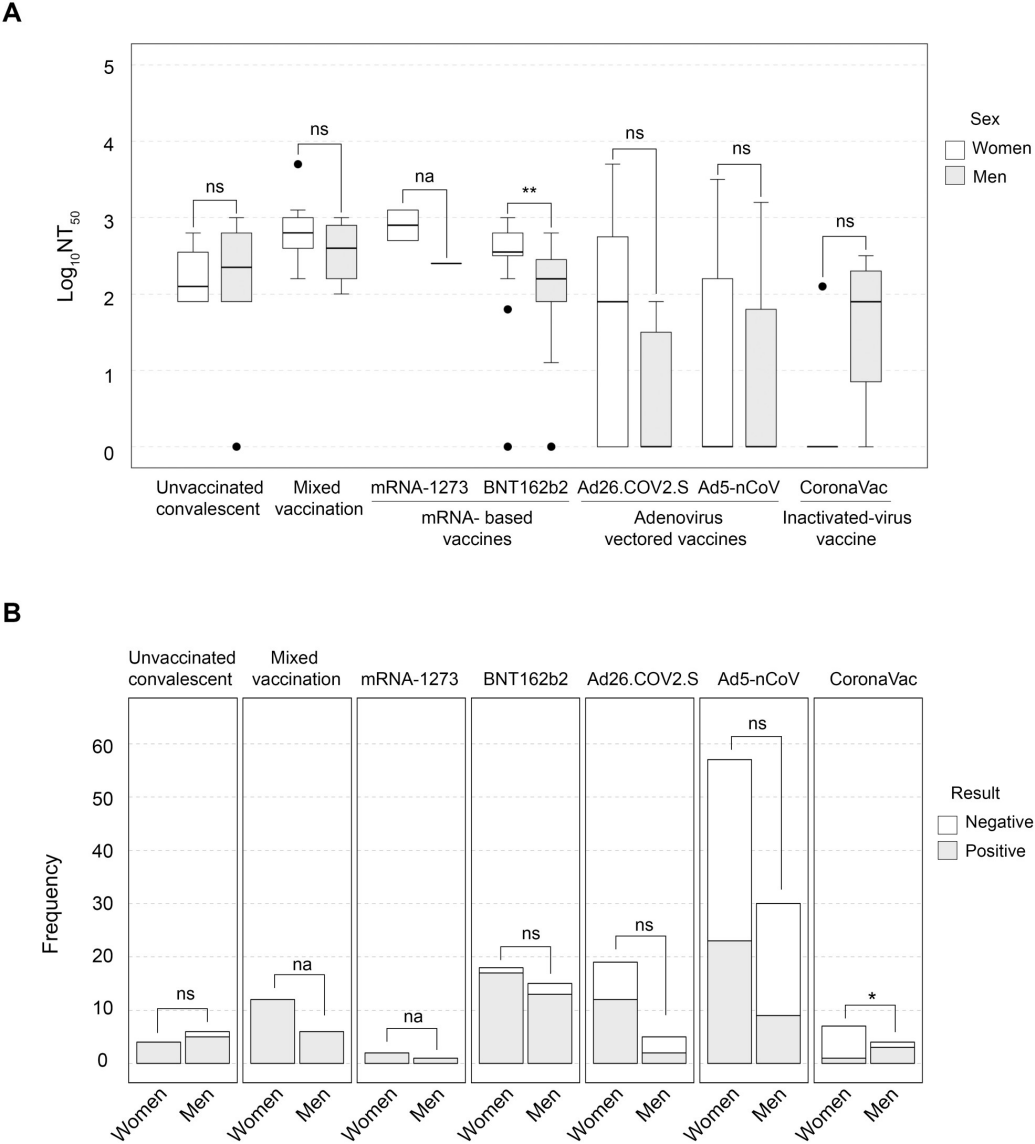
NAb production is similar in women and men among most immunization schemes. A) Box plot showing NAb titers among vaccines grouped by sex. Pairwise comparisons were performed with the Mann-Whitney U test. B) Stacked bars graph showing frequencies in positive and negative results among immunization schemes by sex. Differences between results of women and men were explored by Chi-squared tests. For both panels, na = not applicable for the test, ns = not significant, * = *p* < 0.05, ** = *p* < 0.01.

A previous meta-analysis of vaccine data for SARS-CoV-2 has shown that the efficacy of vaccination is significant for men and women, but when comparing infection rates between individuals, there is a significantly higher rate of protection in males than in females [13]. The immunological response from women and men was also compared in each immunized group, but the only a significant difference with a large effect was found for the BNT162b2 vaccine, from the not infected dataset [U(N_women_ = 18, N_men_ = 15) = 64.50, Z = -2.56, *p* = 0.009, r = -0.45]. In terms of NT_50_, the median for women (249.9) was 2.4-fold higher than for men (103.5). In this analysis, the mRNA-1273 vaccine did not have enough data for performing comparisons (Fig 3A). In contrast, when differences in NAb results were analyzed in the non-infected group by sex, CoronaVac was the only vaccine having a potent and significant effect [*X*^*2*^(1, N = 11) = 4.05, *p* = 0.044, Cramer’s V = 0.60]. Women vaccinated with CoronaVac have a significantly higher rate of negative results (85.71%) than men (25%) (Fig 3B). Also, the Ad26.COV2.S group and unvaccinated convalescents showed a strong association between sex and results were observed, but those differences were not significant (S1 Table, Fig 3B). To validate these effects, a larger sample size would be needed.

### Vaccines schedule protection against SARS-CoV-2 variants

**Fig 4:**
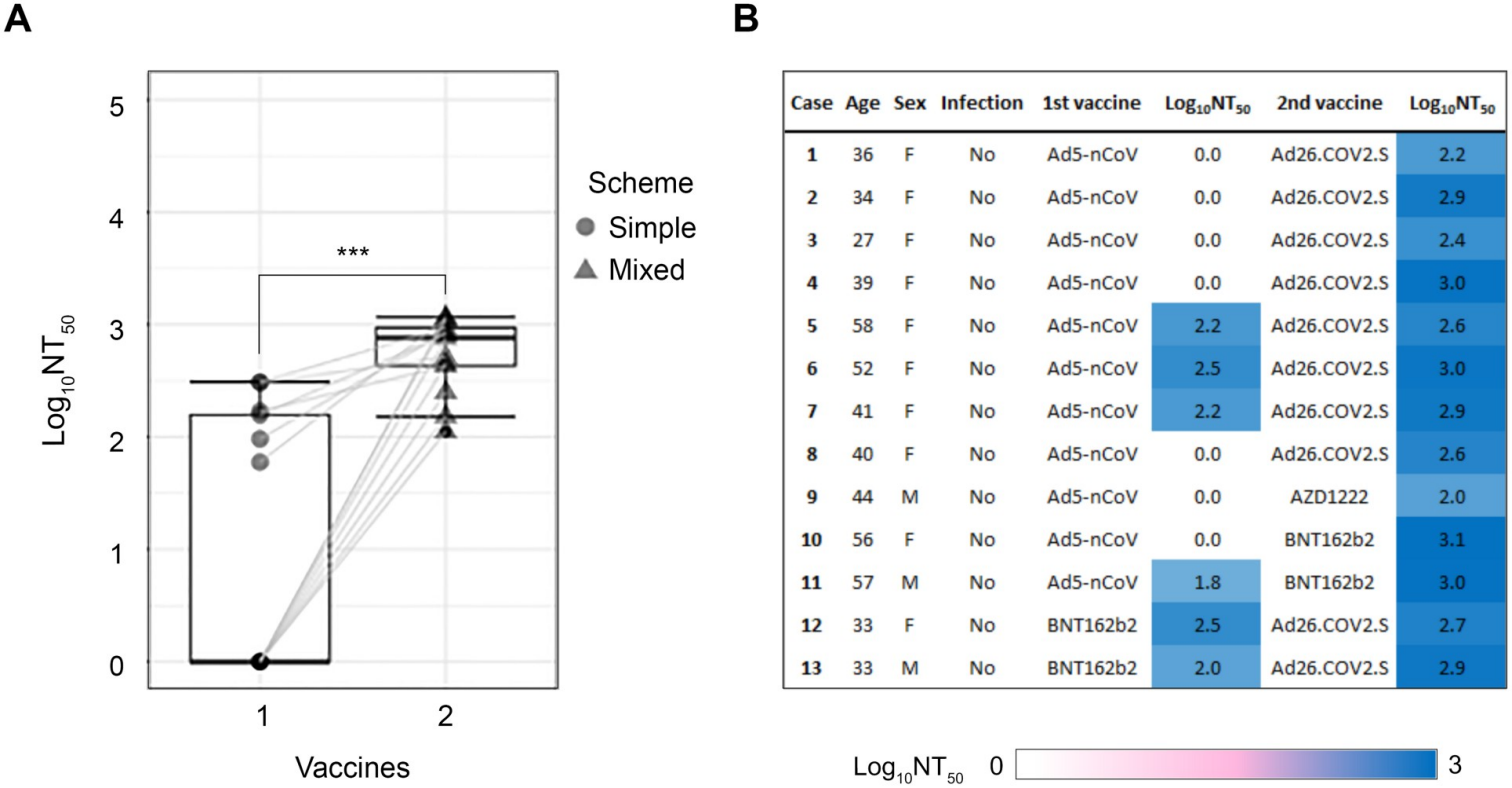
The NAb titers increase in all cases when a second vaccine is received. A) Box plot and tracing of 13 cases before and after mixing vaccines. B) Description of the cases with mixed vaccines. The Log_10_N_50_ titers are highlighted by a color scale from white to blue, corresponding from the lowest to the highest NAb production.

New and highly transmissible SARS-CoV-2 variants with mutations in the S gene are spreading globally and are a major concern in the vaccine effectiveness and vaccination rates among the population [14]. Mixing different Covid-19 vaccines has emerged as a potential solution to protect against emerging variants and increase protection in immunocompromised or individuals with comorbidities. In this context, a preprint study [15] showed that a heterologous schedule of vaccines had proven efficacy against COVID-19 and hospitalization by boosting SARS-CoV-2 anti-spike IgG levels. In our study, 21 volunteers had a confirmed double vaccination scheme; first/second immunization combinations included: Ad5-nCov/Ad2-6.COV2-S (66.67%); Ad5-nCov/Covishield (9.52%); other combinations such as Ad5-nCov/BNT162b2; BNT162b2/Ad2-6.COV2-S, and BNT162b2/AZD1222 Covishield (with 9.52% each). From this mixed vaccination group, we identified 13 volunteers with a previous simple scheme (1-dose vaccine) and a second vaccination scheme (1 or 2-dose vaccine) performed at least 40 days after the first one; all of them with no previous SARS-CoV-2 infection. So, we conducted a paired analysis using the NAb results before and after mixing vaccines, and we found a strong association between positive results and mixing vaccines [*X*^*2*^(1, N = 26) = 9.57, *p* = 0.002, Cramer’s V = 0.60] (Fig 3A). Interestingly, 7 cases had a negative result with a single scheme, but after mixing schemes, production of NAb increased from 2 up to 3 Log_10_NT_50_ [*U*(N_single_ = 113, N_mix_ = 113) = 159.50, Z = 3.89, *p* = 0.00001, r = 0.83] (Fig 4). None of them reported serious adverse effects.

### Vaccinated individuals generate neutralizing antibodies against variants

**Fig 5:**
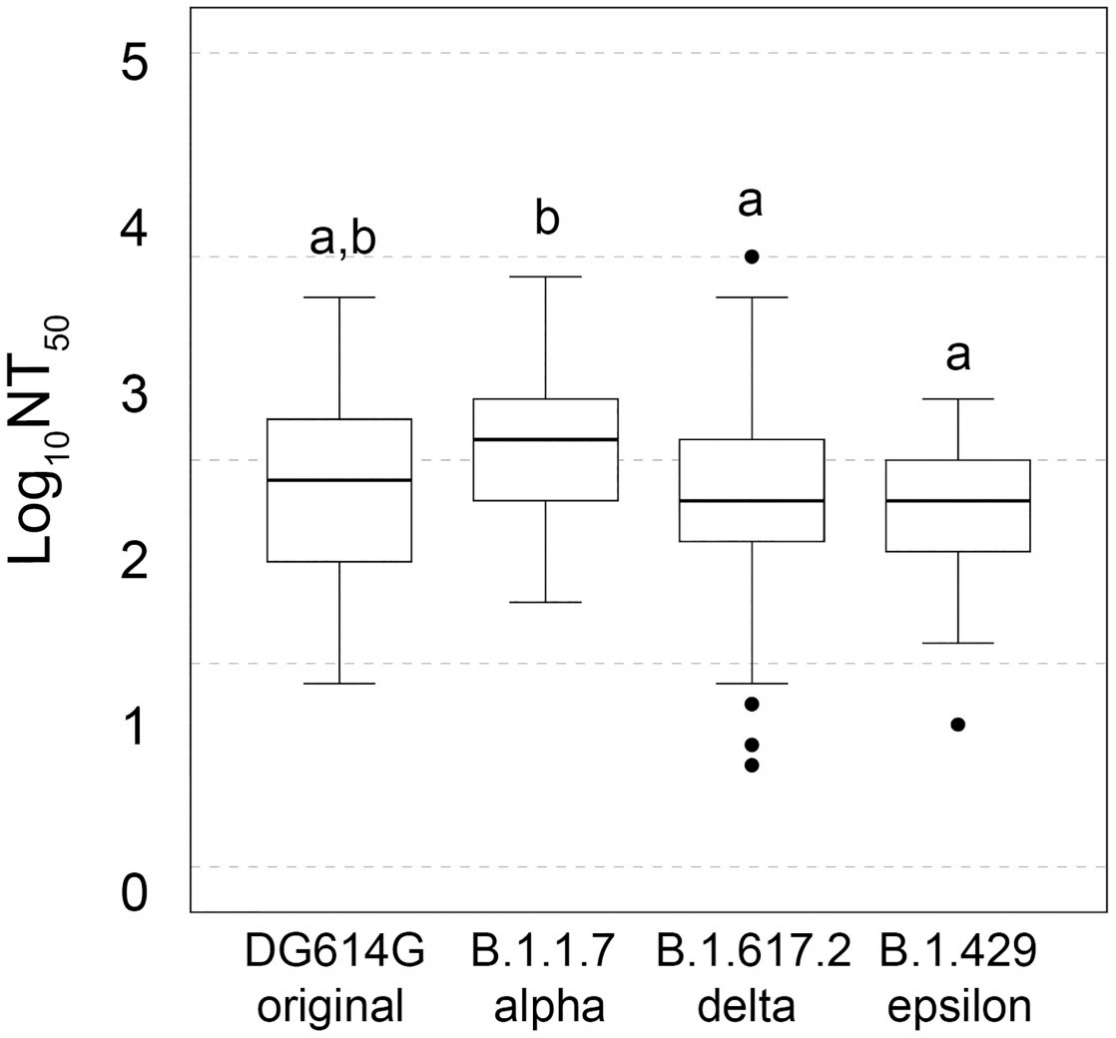
Neutralizing effect of selected sera among four variants. Box plot of NAb titers among variants. Subsets were established by Kruskal–Wallis followed by a post hoc Dunn’s test (adjusted alpha = 0.007). Different letters above bars denote significant differences between groups; the same letter indicates that they belong to the same subset.

Mutations in spike protein with a significant impact on transmissibility, severity, and/or immunity that is likely impact on the epidemiological situation are considered as variants of concern (VOC). Amongst these variants, the B.1.617.2 (delta) variant was the dominant reported variant worldwide in 2021 [16]. Different reports have indicated that members of the B.1.617 lineage exhibit reduced sensitivity to certain monoclonal and polyclonal antibodies [17, 18] and the circulating antibodies of convalescent non-vaccinated individuals [14]. From the total data of previously infected and non-infected individuals obtained against the D614G variant, we selected those plasmas with NT_50_ titers >160 (n = 104) to analyze the neutralization capacity of vaccinated plasma against the viral isolates MEX-BC10/2021 (epsilon B.1.427/B.1.429); MEX-BC12/2021 (alpha B.1.1.7) and MEX-BC15/2021 (delta B.1.617.2). As compared with the neutralization of MX-BC2/2020 (D614G). The analysis of variance, determined significant differences in NAb production among SARS-Cov-2 variants [*H*(3) = 14.75, *p* = 0.002, ε^2^ = 0.15]. Accordingly to Dunn’s test, epsilon and delta variants were the less neutralized by tested sera, followed by D614G, while the alpha variant was the more sensitive to neutralization by plasma of vaccinated individuals regardless of previous SARS-CoV-2 infection (Fig 5) [ACG9]. These results are in line with previous reports where the plasma of vaccinated-convalescent individuals has a maintained but reduced neutralization capacity of B.1.617 variant [14, 17, 19–21] but maintain potency against the B.1.1.7 variant [22]. When we explored differences by vaccine on each variant, none of the comparisons were significant (S2 Fig).

## Discussion

From the onset of the SARS-CoV-2 pandemic, the approval, availability, and use of medical countermeasures, including vaccines to prevent the spread of SARS-CoV-2, have become one of the few effective measure available to slow transmission in the absence of reliable pharmaceutical interventions. This study aimed to evaluate the efficacy of vaccination by measuring the presence of neutralizing antibodies in individuals who received different vaccines in Baja California, Mexico.

According to SARS-CoV-2 patient statistics, for the total of positive cases confirmed in Mexico, there is a predominant percentage of SARS-CoV-2 males compared to women (50.10% vs 49.90%, respectively). In our study, sex-based differences were only significant for the BNT162b2 vaccine, where women produced a significantly higher titer of Nab. These higher antibodies responses in females compared to males have been reported to be higher though not significant in response to SARS-CoV-2 infection, where the outcome of the disease is more negative in male patients [23]. A more robust immunological response to immunization has been reported for influenza vaccines, where females develop an overall immune response that is better males [24].

In accordance with by Lang *et al*. [25], our results showed that the presence and magnitude of NAb were much more frequent and higher in individuals with a previous infection. We did not identify the level of SARS-CoV-2-specific IgM, IgG, and IgA antibodies necessary to predict human population immunity induced by vaccination or previous infection.

It has been reported that variants with the D614G and L425R mutations are more infectious and more resistant to Nab elicited by the original virus or vaccination. Therefore, some studies have reported that the effectiveness of the vaccines against alpha, beta, and delta variants is decreased. [18, 22, 26, 27]. However, in our study, the original virus, delta, epsilon, and alpha variant, remained sensitive to Nab present in the plasma of vaccinated and or convalescent individuals. Our results showed that the delta variant was less sensitive to neutralization by plasma of vaccinated individuals than the D614G strain; at the same time, vaccination conferred a more potent response against than the alpha variant. This result is congruent with the analysis reported by Shen et al. [28], where the alpha variant remains susceptible to Nab elicited by the original virus.

The protection given by vaccines takes time to develop. In addition, people must take all the required doses of a vaccine to build full immunity against the pathogen. For two-dose schedules, vaccines only give partial protection after the first dose, while the second dose increases that protection. With a one-dose vaccine, people will develop maximum immunity against SARS-CoV-2 a few weeks after getting vaccinated. Their efficacy by reducing the relative risk of infection becomes the primary target for their overall approval. Here we show that vaccines with a double scheme based on mRNA seem to be more efficient in promoting the development of NAb. Since neutralization is the ability of antibodies to block virus infection, our results indicate that mixed vaccination induces the highest immunological response, followed by SARS-CoV-2 infection and mRNA-based vaccines immunization either in a single or double dose; vaccination with adenovirus-based vaccines and inactivated-viral particles were the less efficient in promoting the generation of NAbs. We showed that infection- and vaccine-induced immunity might be retained against SARS-CoV-2 and increased by mixing vaccination schedules giving the immune system better ways to recognize and neutralize the pathogen. While vector-based vaccines promote a better T-cell response [29], the mRNA-based vaccines generate a strong antibody response by promoting the generation of Circulating IgG- and IgA-secreting plasmablasts that target the S protein [30]. Both characteristics are needed to reach a robust immune response and achieve long-term protection against disease and overall mortality. Xinxue Liu and colleagues [15], showed that the immunological response of double-dose ChAdOx1 nCoV-19 (AstraZeneca) is statistically lower than schedules including BNT162b2 and ChAdOx1 at 28 days post-boost dose; suggesting that a heterologous schedule based on the sequential administration both vaccines could be highly immunogenic, and perhaps more than homologous schedules. In a similar study performed by Borobia et al., [31], described by Callaway [32], people who received a dose of the BNT162b2 vaccine eight weeks after an initial AZD1222 dose had few side effects and a robust antibody response two weeks after the second shot. This analysis was restricted to both vaccines and the main response seems to be influenced after the first neutralization. In our study, a previous Ad5-nCoV single schedule was followed by a single heterologous immunization with Ad2-6.COV2-S induced a strong antibody response even after 60 days post immunization; both schemes are adeno vector-based vaccines with no previous report of heterologous use. For the combination of mRNA-based followed by vector-based vaccination or vice versa, the results were similar, indicating that a robust immune response can be induced by a heterologous scheme regardless of the previous vaccination; and that the heterologous vaccination scheme could be considered, in some circumstances, for the national vaccine program in Mexico. Antibodies perform a fundamental role against infections and protect cells by directly blocking virus entrance; different vaccination schemes around the globe have pursued their function in protection; nonetheless, antibody persistence needs constant boosting of doses over time. Accumulating evidence of highly persistent antibodies generated by heterologous vaccinations has been documented [33–35]: also, there is evidence that heterologous schemes promote CD4+ and CD8+ reactive T cells against different variants [35]. Amplifying the immunological responses of the population against variants by promoting heterologous schemes can be a solution to raise persistent T cells responses and highly specialized antibodies capable of recognizing different spike variants. Also, for herd immunity purposes, we need to consider the hesitation of the population to vaccination; in the review performed by T.C Ho *et al*. [33], the presence of uncommon serious adverse events is found in the immunization with ChAdOx1-S, Ad26.COV2-S, BNT162b2, and mRNA-1273, leading to a reduced number of fully vaccinated subjects, besides the overall vaccine availability and population confidence in different brands. As mentioned by these authors, the more important studies about heterologous vaccination have been made in Europe, focused on using ChAdOx1-S as the primary dose, followed by mRNA schemes. Our findings support the studies of heterologous schemes by including data on different types of vaccines as primary or secondary boosts regardless of their type. In all papers where heterologous vaccination has been studied or summarized, the increase of antibody titers has been a constant improvement, but also, besides the increased antibody titers elicited by vaccination with BNT162b2 or ChAdOx1; we need to consider that the overall protection of both vaccines has been associated with lower viral loads in Delta and Alpha variants. Nonetheless, reductions in transmission are lower for the Delta variant than Alpha for BNT162b2 vaccination [36].

Our results showed that vaccinated individuals with previous SARS-CoV-2 infection had the highest titers of NAbs against tested variants. In this context, hybrid immunity might also be responsible for falling case numbers across countries with higher infection rates. About 90% of the neutralizing activity present in SARS-CoV-2 immune sera by NAb responses elicited during natural infection are directed against the receptor-binding domain (RBD) [36]. Nonetheless, a study performed by Iyer et al. [37] showed that anti-RBD IgG responses decayed slowly through 90 days, and mutations found in SARS-CoV-2 variants have been shown to decrease sensitivity to neutralization by monoclonal antibodies, convalescent plasma, and sera from vaccinated individuals [38]. Scaping neutralization by circulating antibodies generated either by a previous infection or vaccination represents a significant concern in the efforts to contain the SARS-CoV-2 globally; in this context, our results along with other studies have shown that hybrid immunity promotes potent and broadly neutralizing antibodies [38–40]. All these data suggest that in people seropositive to SARS-CoV-2, vaccination will produce antibodies with increased potency and will be able to control SARS-CoV-2 emerging variants better.; At the same time, in those individuals with no previous history of Covid-19, a third vaccine dose might allow achieving the benefits of hybrid immunity.

A previous study by Schmidt *et al*. [41] showed that priming with a vector-based vaccine followed by a mRNA vaccine induce higher neutralizing antibodies and spike-specific CD4 T cell responses than homologous vector or mRNA vaccine schedules in healthy individuals. Our results showed that heterologous vaccination with an adenovirus-based vaccine, followed by a second vector-based or mRNA-based vaccine, induces higher antibody titers regardless of initial priming. Indicating that combining different technologies is a good strategy for reinforce immunity; besides this, a previous report by Gram *et al*. [34], showed that there’s a reduction in the risk of SARS-CoV-2 infection when combining the ChAdOx1 and an mRNA vaccine in the population when compared with unvaccinated individuals. According to these studies, our results re-emphasize the urgent need to improve effective vaccine strategies in order to provide broader population protection against the emerging VOC of SARS-CoV-2. Our findings clearly show that weaker responses generated, for example, by natural infection or single doses vaccines, can be overcome by modifying the single schemes to mixed ones or by providing a second or third dose to the general population, providing a reinforced neutralization of virus by circulating antibodies. Governments can install heterologous vaccination as a strategy to overcome low vaccines availability.

Restrictions in this study include the follow-up of participants after 60 days post-immunization to measure long-term antibody responses, along with the reduced number of participants needed to extrapolate results to the general Mexican population.

## Conclusion

Overall, in our study, all analyzed vaccines generate neutralizing antibodies that may reduce the risk of SARS-CoV-2 infection and severe COVID-19. With the BNT162b2 vaccine show the capacity to induces the highest levels of neutralizing antibodies. Individuals with no history of SARS-CoV-2 infection and vaccinated with Ad5-nCoV generated lower levels of detectable neutralizing antibodies. However, when previously infected individuals were vaccinated with Ad5-nCoV, the neutralizing antibodies increased to levels that resembled a second dose vaccine, indicating that a second boost may be necessary for Ad5-nCoV.

Slight differences in vaccine effectiveness were noted between delta, alpha, and D614G variants (ε2 <0.2) and large effect (r >0.4) was observed in vaccine effectiveness on convalescent individuals. Plasmas with detectable NAbs levels neutralized all variants from 1:40 titers. Since the minimum titer of SARS-CoV-2 protein S neutralizing antibodies to induce protection is unknown and the neutralization titers do not measure all protected levels induced by a single vaccine, these results are suggestive of protection in the analyzed population and do not substitute the vaccine efficacy of the cellular response.

In a mixed scheme, the second dose with Ad5-nCoV (CanSino) induced a robust immune response compared to those with a single dose. The protection induced by a heterologous boost is equivalent to those vaccines with higher NAbs titers. This is the first report analyzing the efficacy of mixing the Ad5-nCov vaccine with other immunizations schemes.

Considering a SARS-CoV-2 infection as a prime contact of the virus with the patient’s immune system (natural immunization) and the development of higher titers in a single prime-boost, we can consider that a second or even a third boost may help develop a robust neutralizing antibodies response in the general population.

## Supporting information

S1 Table Statistical analyses performed during the study. (DOCX)

S1 Fig Exploratory analysis of variables influencing NAb production. A) Violin plot highlighting significant differences between not infected and convalescents. B) Violin plot denoting similar densities in both sexes. Pairwise comparisons in panels A-B were performed with Mann-Whitney U test where ns = not significant, *** = *p* < 0.001. C) Dispersion plot of NAb titers. The line indicates the linear correlation between age and NAb titers, where R^2^ is the square of the correlation coefficient.
(TIF)

S2 Fig Immunization schemes were not differential among variants. Box plot of NAb titers from variants by vaccine. Only immunization schemes with enough data were plotted and analyzed by Kruskal–Wallis test. ns = no significance (p > 0.05).
(TIF)
