# Supplementary material for: Neutralizing antibodies levels are increased in individuals with heterologous vaccination and hybrid immunity with Ad5-nCoV in the north of Mexico
Source: PLoS One. 2022 Jun 24;17(6):e0269032. doi: 10.1371/journal.pone.0269032 (PMC9231729; doi:10.1371/journal.pone.0269032)
Supplement: S1 Table — (DOCX) [file pone.0269032.s001.docx]

**S1 Table. Statistical analyses performed during the study**

| **Stage of the analysis** | **Data set** | **n** | **Independent variable(s)** | **Dependent variable** | **Analysis** | **Result** | **Figure** |
| --- | --- | --- | --- | --- | --- | --- | --- |
| First-exploratory | All cases | 263 cases | NA | NT_50_ | Shapiro-Wilk test | W(265)= 0.64, p= 2.88E-23 | NA |
| First-exploratory | All cases | 263 cases | NA | Log_10_NT_50_ | Shapiro-Wilk test | W(265)= 0.83, p= 1.85E-16 | NA |
| First-exploratory | All volunteers | 244 volunteers | Age | NA | Descriptive statistics | Mean 43.06, Median 40.5, Mode 54, SD 13.26, Min. 19, Máx. 82, Rank 63, | NA |
| First-exploratory | All cases | 263 cases | Years | Log_10_NT_50_ | Bivariate Pearson correlation, linear regresion | R(260)= -0.036961, p= 0.550674, R2= 0.001 | NA |
| First-exploratory | All cases | 263 cases | Result (positive, negative) | Years | Mann-Whitney U test, rank biserial | U(N_negative_= 89, N_positive_= 174)= 7989.5, Z= 0.422489, p= 0.672668 | NA |
| First-exploratory | Vaccinated people | 245 cases | Years | Log_10_NT_50_ | Bivariate Pearson correlation, linear regresion | R(244)= -0.069155, p= 0.280944, R2= 0.004 | S1C Figure |
| First-exploratory | Vaccinated people | 245 cases | Years | Result (positive, negative) | Mann-Whitney U test, rank biserial | U(N_negative_= 85, N_positive_= 160)= 7186.0, Z= 0.731330, p= 0.464578, r= 0.05 | NA |
| First-exploratory | Vaccinated people | 247 cases | Sex | Log_10_NT_50_ | Mann-Whitney U test, rank biserial | U(N_women_= 157, N_men_= 90)= 6155.500000, Z= -1.720545, p= 0.085333, r= -0.11 | S1B Figure |
| First-exploratory | Vaccinated people | 247 cases | Sex | Result (positive, negative) | Chi-Square Test, Phi | X^2^(1, N=247)= 1.034017, p= 0.309217, ϕ 0.064702 | NA |
| First-exploratory | Vaccinated people | 242 cases | Infection | Log_10_NT_50_ | Mann-Whitney U test, rank biserial | U(Nnot infected= 176, Nconvalescent= 66)= 2270.500, Z = -7.454815, p= 9.015E-14, r = -0.48 | S1A Figure |
| First-exploratory | Vaccinated people | 242 cases | Infection | Result (positive, negative) | Chi-Square Test, Phi | X^2^(1, N=242)= 17.784867, p= 0.000025, ϕ 0.271093 | NA |
| First-exploratory | Mixed vaccination | 20 cases | Infection | Log_10_NT_50_ | Mann-Whitney U test, rank biserial | U(Nnot infected= 18, Nconvalescent= 2)= 2.50 , Z = -1.963923, p= 0.042105, r = 0.44 | Figure 1A |
| First-exploratory | mRNA-1273 vaccine | 8 cases | Infection | Log_10_NT_50_ | Mann-Whitney U test, rank biserial | U(Nnot infected= 3, Nconvalescent= 5)= 2.000, Z = -1.680278, p= 0.142857, r = -0.59 | Figure 1A |
| First-exploratory | BNT162b2 | 49 cases | Infection | Log_10_NT_50_ | Mann-Whitney U test, rank biserial | U(Nnot infected= 16, Nconvalescent= 33)= 48.00, Z= -4.616337 , p= 0.000004 , r = -0.66 | Figure 1A |
| First-exploratory | Ad26.COV2.S | 36 | Infection | Log_10_NT_50_ | Mann-Whitney U test, rank biserial | U(Nnot infected= 24, Nconvalescent= 12)= , Z = -2.583989, p=0.009819 , r = -0.43 | Figure 1A |
| First-exploratory | Ad5-nCoV | 110 | Infection | Log_10_NT_50_ | Mann-Whitney U test, rank biserial | U(Nnot infected= 87, Nconvalescent= 33)= 375.500000, Z= -4.997014, p= 5.8225E-7, r = -0.48 | Figure 1A |
| First-exploratory | CoronaVac | 19 | Infection | Log_10_NT_50_ | Mann-Whitney U test, rank biserial | U(Nnot infected= 11, Nconvalescent= 8)= 17.500000, Z = -2.315574, p= 0.025932, r = -0.53 | Figure 1A |
| First-exploratory | Mixed vaccination | 20 cases | Infection | Result (positive, negative) | Chi-Square Test, Cramer's V | Statistics were not calculated because all results are positive | Figure 1B |
| First-exploratory | mRNA-1273 vaccine | 8 cases | Infection | Result (positive, negative) | Chi-Square Test, Cramer's V | Statistics were not calculated because all results are positive | Figure 1B |
| First-exploratory | BNT162b2 | 49 cases | Infection | Result (positive, negative) | Chi-Square Test, Cramer's V | X^2^(1, N=49)= 0.116004, p= 0.733410, Cramer's V= 0.048656 | Figure 1B |
| First-exploratory | Ad26.COV2.S | 36 | Infection | Result (positive, negative) | Chi-Square Test, Cramer's V | X^2^(1, N=36)= 2.250000, p= 0.133614, Cramer's V=0.250000 | Figure 1B |
| First-exploratory | Ad5-nCoV | 110 | Infection | Result (positive, negative) | Chi-Square Test, Cramer's V | X^2^(1, N=110)= 15.362507, p= 0.000089, Cramer's V= 0.373710 | Figure 1B |
| First-exploratory | CoronaVac | 19 | Infection | Result (positive, negative) | Chi-Square Test, Cramer's V | X^2^(1, N=19)= 2.773232, p= 0.095852, Cramer's V= 0.382047 | Figure 1B |
| First-exploratory | BNT162b2 | 58 cases | Infection | Log_10_NT_50_ | Mann-Whitney U test, rank biserial | U(Nnot_infected= 38, Nconvalescent= 20)= 77.500000, Z= -4.960679, p= 7.0247E-7, r = -0.64 | Figure 1C |
| First-exploratory | BNT162b2 not infected | 38 cases | Scheme | Log_10_NT_50_ | Mann-Whitney U test, rank biserial | U(N_1-dose_= 5, N_2-doses_= 33)= 18.500000, Z= -2.779749, p= 0.003064, r = -0.45 | Figure 1C |
| First-exploratory | BNT162b2 convalescent | 20 casses | Scheme | Log_10_NT_50_ | Mann-Whitney U test, rank biserial | U(N_1-dose_= 4, N_2-doses_= 16)= 163.500000, Z= -1.227095, p= 0.219787, r = | Figure 1C |
| First-exploratory | BNT162b2 | 58 cases | Infection | Log_10_NT_50_ | Kruskal–Wallis test, epsilon squared, post-hoc Dunn's test with Bonferroni correction | *H*(3)= 28.669347, p= 0.000003, ε^2^= 0.591175, post-hoc Dunn's test (adjusted alpha 0.0125) | Figure 1D |
| Second-immunization schemes | convalescent | 77 cases | Vaccines | Log_10_NT_50_ | Kruskal–Wallis test, epsilon squared, post-hoc Dunn's test with Bonferroni correction | *H*(6)= 16.648746, p= 0.010665, ε^2^= 0.559532, post-hoc Dunn's test (adjusted alpha 0.007) | Figure 2A |
| Second-immunization schemes | convalescent | 77 cases | Vaccines | Result (positive, negative) | Chi-Square Test, Cramer's V | X^2^(6, N=77)= 3.443313, p= 0.751496, Cramer's V= 0.211467 | NA |
| Second-immunization schemes | Unvaccinated convalescent, vaccinated not infected | 186 cases | Vaccines | Log_10_NT_50_ | Kruskal–Wallis test, post-hoc Dunn's test with Bonferroni correction, epsilon squared | *H*(6)= 61.435, p=2.2995E-11 , ε2= 0.524449, post-hoc Dunn's test (adjusted alpha 0.007) | Figure 2B |
| Second-immunization schemes | Unvaccinated convalescent, vaccinated not infected | 186 cases | Vaccines | Result (positive, negative) | Chi-Square Test, Cramer's V | X^2^(6, N=186)= 52.598711, p= 1.4132E-9, Cramer's V= 0.531779 | NA |
| Second-immunization schemes | Vaccinaed convalescent | 66 cases | Sex | Result (positive, negative) | Chi-Square Test, Cramer's V | X^2^(1, N=66)= 3.670244, p= 0.055392, Cramer's V= 0.235817 | NA |
| Second-immunization schemes | Vaccinaed convalescent | 66 cases | Sex | Log_10_NT_50_ | Mann-Whitney U test, rank biserial | U(N_women_= 41, N_men_= 25)= 507.000000, Z= -0.073023, p= 0.941788, r = -0.01 | NA |
| Second-immunization schemes | Vaccinaed not infected | 176 cases | Sex | Result (positive, negative) | Chi-Square Test, Cramer's V | X^2^(1, N=176)= 0.103765, p= 0.747358, Cramer's V= 0.024281 | NA |
| Second-immunization schemes | Vaccinaed not infected | 176 cases | Sex | Log_10_NT_50_ | Mann-Whitney U test, rank biserial | U(N_women_= 115, N_men_= 61)= 3011.500000, Z= -1.606173, p= 0.108236, r = -0.12 | NA |
| Second-immunization schemes | Unvaccinated convalescent | 10 cases | Sex | Log_10_NT_50_ | Mann-Whitney U test, rank biserial | U(N_women_= 4, N_men_= 6)= 14.000000, Z= 0.434372, p= 0.761905, r= 0.14 | Figure 3A |
| Second-immunization schemes | Not infected vaccinated with Mix | 18 cases | Sex | Log_10_NT_50_ | Mann-Whitney U test, rank biserial | U(N_women_= 12, N_men_= 6)= 23.500000, Z= -1.179282, p= .250, r= -0.28 | Figure 3A |
| Second-immunization schemes | Not infected vaccinated with BNT162b2 | 33 cases | Sex | Log_10_NT_50_ | Mann-Whitney U test, rank biserial | U(N_women_= 18, N_men_= 15)= 64.500000, Z= -2.564407, p= 0.009432, r= -0.45 | Figure 3A |
| Second-immunization schemes | Not infected vaccinated with mRNA-1273 | 8 cases | Sex | Log_10_NT_50_ | Mann-Whitney U test, rank biserial | Statistics were not calculated because there are less than 3 cases per group | Figure 3A |
| Second-immunization schemes | Not infected vaccinated with Ad26.COV2.S | 24 cases | Sex | Log_10_NT_50_ | Mann-Whitney U test, rank biserial | U(N_women_= 19, N_men_= 5)= 28.000000, Z= -1.440348, p= 0.182900, r= -0.29 | Figure 3A |
| Second-immunization schemes | Not infected vaccinated with Ad5-nCoV | 87 cases | Sex | Log_10_NT_50_ | Mann-Whitney U test, rank biserial | U(N_women_= 57, N_men_= 30)= 743.500000, Z= -1.152013, p= 0.249316, r= -0.12 | Figure 3A |
| Second-immunization schemes | Not infected vaccinated with CoronaVac | 11 cases | Sex | Log_10_NT_50_ | Mann-Whitney U test, rank biserial | U(N_women_= 7, N_men_= 4)= 22.500, Z= 1.866197, p= 0.109091, r= 0.56 | Figure 3A |
| Second-immunization schemes | Unvaccinated convalescent | 10 cases | Sex | Result (positive, negative) | Chi-Square Test, Cramer's V | X^2^(1, N=10)= 0.740741, p= 0.389424, Cramer's V= 0.272166 | Figure 3B |
| Second-immunization schemes | Not infected vaccinated with Mix | 18 cases | Sex | Result (positive, negative) | Chi-Square Test, Cramer's V | Statistics were not calculated because all results are positive | Figure 3B |
| Second-immunization schemes | Not infected vaccinated with mRNA-1273 | 8 cases | Sex | Result (positive, negative) | Chi-Square Test, Cramer's V | Statistics were not calculated because all results are positive | Figure 3B |
| Second-immunization schemes | Not infected vaccinated with BNT162b2 | 33 cases | Sex | Result (positive, negative) | Chi-Square Test, Cramer's V | X^2^(1, N=33)= 0.598889, p= 0.439002, Cramer's V= 0.134715 | Figure 3B |
| Second-immunization schemes | Not infected vaccinated with Ad26.COV2.S | 24 cases | Sex | Result (positive, negative) | Chi-Square Test, Cramer's V | X^2^(1, N=24)= 0.873383, p= 0.350020, Cramer's V= 0.190764 | Figure 3B |
| Second-immunization schemes | Not infected vaccinated with Ad5-nCoV | 87 cases | Sex | Result (positive, negative) | Chi-Square Test, Cramer's V | X^2^(1, N=87)= 0.905643, p= 0.341273, Cramer's V= 0.102028 | Figure 3B |
| Second-immunization schemes | Not infected vaccinated with CoronaVac | 11 cases | Sex | Result (positive, negative) | Chi-Square Test, Cramer's V | X^2^(1, N=11)=4.054847, p= 0.044045, Cramer's V= 0.607143 | Figure 3B |
| Second-immunization schemes | Mixed vaccination | 13 volunteers | Scheme | Log_10_NT_50_ | Mann-Whitney U test, rank biserial | U(N_single_= 13, mix= 11)= 159.500000, Z= 3.894, p= 0.000019, r= 0.83 | Figure 4A |
| Second-immunization schemes | Mixed vaccination | 13 volunteers | Scheme | Result (positive, negative) | Chi-Square Test, Cramer's V | X^2^(1, N=26)= 9.578947, p= 0.001968, Cramer's V= 0.606977 | NA |
| Third-variants | All variants | 289 cases | NA | Log_10_NT_50_ | Shapiro-Wilk test | W(288)= 0.988409, p= 0.020645 | NA |
| Third-variants | All variants | 289 cases | Variant | Log_10_NT_50_ | Kruskal–Wallis test, epsilon squared, post-hoc Dunn's test with Bonferroni correction | *H*(3)= 14.756443, p= 0.002037, ε^2^= 0.15, post-hoc Dunn's test (adjusted alpha 0.007) | Figure 5 |
| Third-variants | Variants not infected | 61 cases | D614G | Log_10_NT_50_ | Kruskal–Wallis test, epsilon squared | *H*(3)= 9.810627, p= 0.097567, ε2= 0.224171 | S2 Figure |
| Third-variants | Variants convalescent | 35 cases | Alpha | Log_10_NT_50_ | Kruskal–Wallis test, epsilon squared | *H*(3)= 4.744725, p= 0.191473, ε2= 0.251631 | S2 Figure |
| Third-variants | Variants convalescent | 35 cases | Epsilon | Log_10_NT_50_ | Kruskal–Wallis test, epsilon squared | *H*(3)= 3.332, p= 0.343232, ε2= 0.249247 | S2 Figure |
| Third-variants | Variants not infected | 60 cases | Delta | Log_10_NT_50_ | Kruskal–Wallis test, epsilon squared | *H*(3)= 6.647, p= 0.084033, ε2= 0.228808, post-hoc Dunn's test (adjusted alpha 0.007) | S2 Figure |
