# Supplementary figures and images for: Neutralizing antibodies levels are increased in individuals with heterologous vaccination and hybrid immunity with Ad5-nCoV in the north of Mexico
Source: PLoS One. 2022 Jun 24;17(6):e0269032. doi: 10.1371/journal.pone.0269032 (PMC9231729; doi:10.1371/journal.pone.0269032)

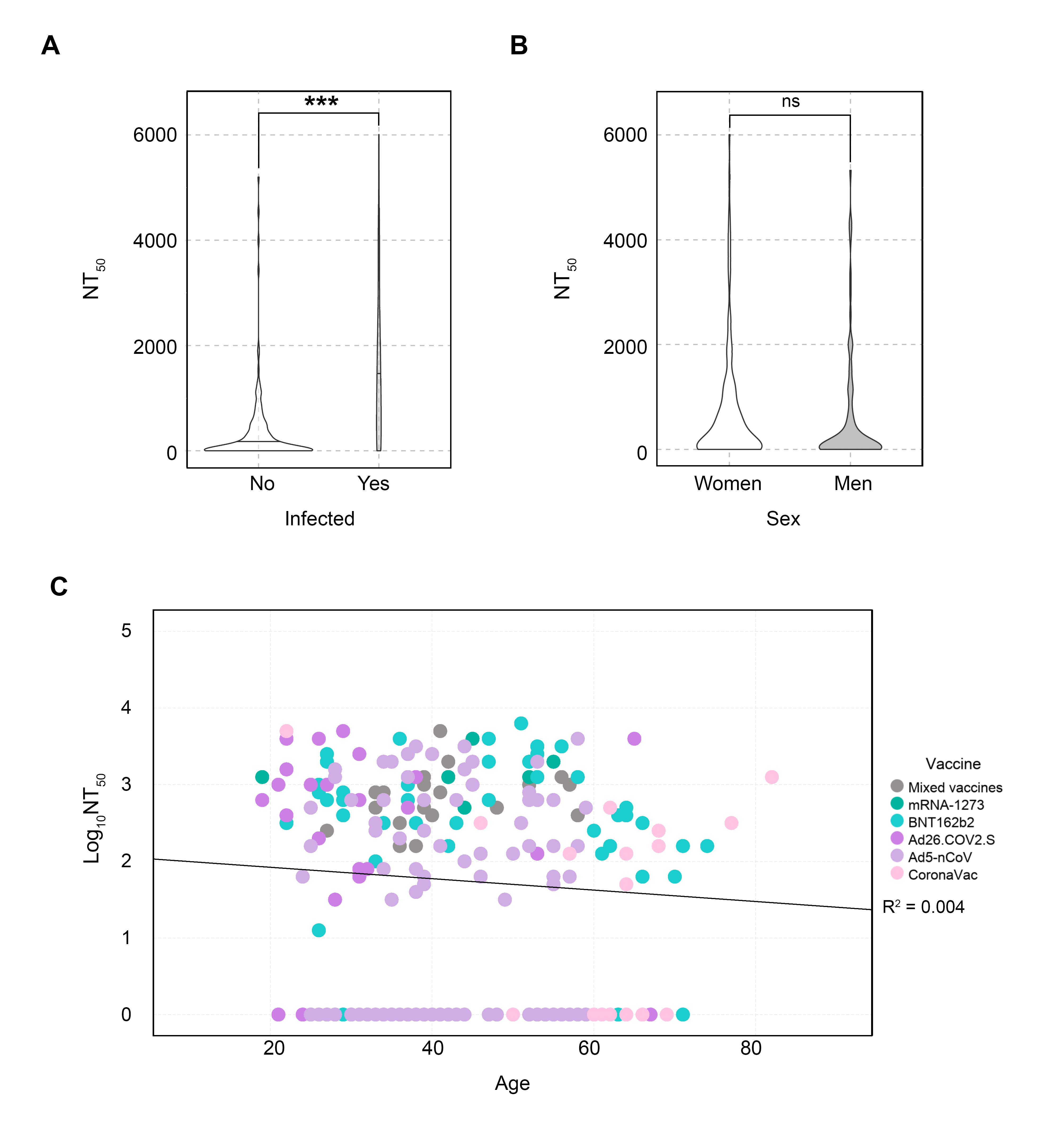

Supplement: S1 Fig — A) Violin plot highlighting significant differences between not infected and convalescents. B) Violin plot denoting similar densities in both sexes. Pairwise comparisons in panels A-B were performed with Mann-Whitney U test where ns = not significant, *** = p < 0.001. C) Dispersion plot of NAb titers. The line indicates the linear correlation between age and NAb titers, where R2 is the square of the correlation coefficient. (TIF) [file pone.0269032.s002.tif]

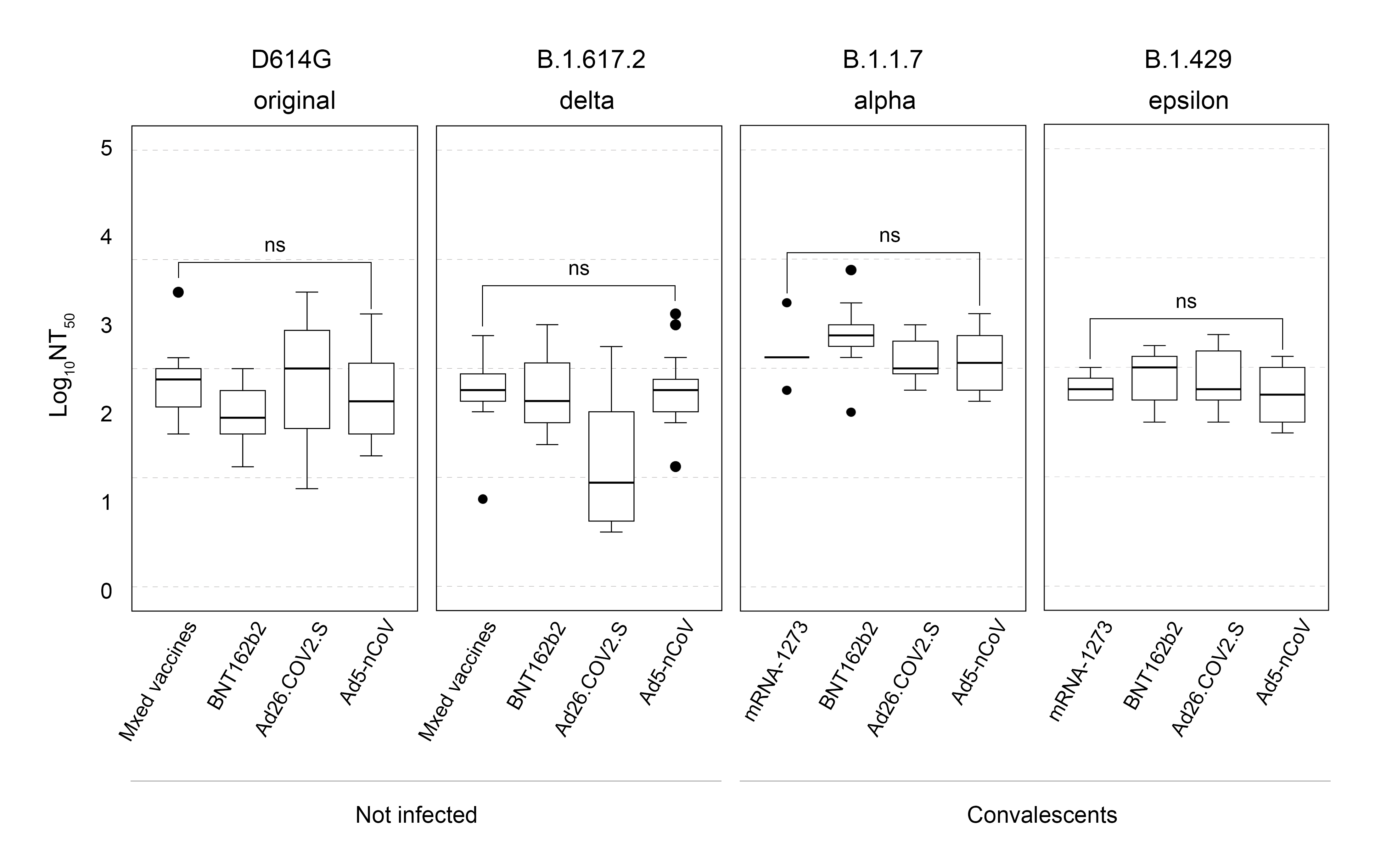

Supplement: S2 Fig — Box plot of NAb titers from variants by vaccine. Only immunization schemes with enough data were plotted and analyzed by Kruskal–Wallis test. ns = no significance (p > 0.05). (TIF) [file pone.0269032.s003.tif]
